# Mapping the global intellectual landscape of inflammatory tumor microenvironment in colorectal cancer pathogenesis and prognostic research since this century

**DOI:** 10.1007/s12672-025-03524-w

**Published:** 2025-10-14

**Authors:** Hui Mu, Yushu Zhu, Feihu Yan, Can Lv, Xiaoyu Tu, Junyan He, Zhaoming Wang, Yi Zeng, Zhiwei Liu, Jiaojiao Chen, Bai Li, Enda Yu, Xuan Zheng

**Affiliations:** 1 Department of Clinical Nutrition, The First Affiliated Hospital of Naval Medical University, Shanghai, 200433 China; 2 School of Traditional Chinese Medicine, Naval Medical University, Shanghai, 200433 China; 3 Department of Burn Surgery, The First Affiliated Hospital of Naval Medical University, Shanghai, 200433 China; 4 Department of Colorectal Surgery, Changhai Hospital, Naval Medical University, Shanghai, 200433 China; 5 Department of Rehabilitation Medicine and Physiotherapy, First Affiliated Hospital of Naval Medical University, Shanghai, 200433 China; 6 Department of Chinese Medicine and Oncology, Changhai Hospital, Naval Medical University, Shanghai, 200433 China

**Keywords:** Colorectal cancer, Inflammation, Tumor microenvironment

## Abstract

**Background:**

Colorectal cancer (CRC) is one of the most prevalent gastrointestinal tumors worldwide. Complex interactions between tumor microenvironment (TME) and host inflammatory response influence CRC progression and recurrence. The current study provides a comprehensive bibliometric visualization of the current state and frontier trends of inflammatory TME in CRC.

**Materials and methods:**

Scientific publications on the inflammatory TME in CRC from 2000 to 2024 were retrieved from the Web of Science Core Collection (WoSCC) database. Biblioshiny software was mainly applied for visualization and analysis of literature. CiteSpace software and VOSviewer software were used to validate the results.

**Results:**

A total of 1593 articles related to CRC inflammatory TME have been retrieved since the 21st century. *Cancers* and *Frontiers in Immunology* were the two most popular journals, and *Cancer Research* is the most cited journal. Mcmillan DC, Park JH and Mantovani A were the most academic influential authors in inflammatory TME in CRC.

**Conclusions:**

This study preliminary visualize the association of inflammatory TME with CRC through bibliometric analysis. The immunomodulatory mechanisms in IBD-associated carcinogenesis, NF-κB-mediated TME remodeling in tumor progression, and CRC patient stratification and precision therapeutics were three hotspots for future research endeavors on the inflammatory TME in CRC research.

**Supplementary Information:**

The online version contains supplementary material available at 10.1007/s12672-025-03524-w.

## Introduction

Colorectal cancer (CRC) stands as one of the most prevalent malignancies affecting the digestive tract globally [1]. The incidence of CRC has been progressively rising, largely due to alterations in social and dietary patterns, particularly impacting younger demographics [2, 3]. As reported by the International Agency for Research on Cancer, in 2020, there were 1.93 million novel cases and 935,000 fatalities worldwide attributed to CRC [1]. CRC patients often experience deregulated signaling pathways leading to tumor cell detachment, invasion, and metastasis, threatening their lives [4]. This process ultimately culminates in invasive CRC metastasis to distant organs, further threatening patients’ lives. It is noteworthy that approximately 25% of CRC patients exhibit metastasis at the time of diagnosis [5]. Radical surgical resection of cancerous lesions remains the most efficacious treatment for CRC [6], yet its effectiveness is restricted to patients without tumor metastasis, and recurrence is not precluded following surgery [7]. While current treatment modalities, including chemotherapy and immunotherapy, have shown some enhancement in survival rates [8, 9], a comprehensive and satisfactory treatment regimen remains elusive.

The complexity of CRC treatment lies in the intricate interplay between the tumor microenvironment (TME) and the inflammatory response of the host, influenced by aberrant cancer cells [10]. Stephen Paget’s “seed and soil” hypothesis, first introduced in 1889, underscored the significance of the TME in cancer development [11]. Research has expanded our understanding of the TME, which includes cellular and non-cellular components that shield tumor cells from apoptosis and immune surveillance, promoting growth, progression, and metastasis [12–14]. Typically, the inflammatory processes involved in tissue repair subside autonomously after tissue regenerative repair and pathogen elimination. However, this is not always the case, as chronic inflammation releases a variety of biologically active substances, produces inflammatory TME that facilitates cancer cell growth and malignant transformation of stromal cells and inhibits the restoration of normal homeostasis of the body’s tissues, thereby inducing local tissue tumorigenesis and metastasis [4]. Recently, the growth of bioinformatics and technologies, including second-generation and single-cell sequencing, has revolutionized our investigation into the pathophysiology of the inflammatory TME and its role in tumor development and metastasis [15].

Bibliometrics, a research methodology that leverages statistics and mathematics, offers a comprehensive quantitative and qualitative analysis of the network structure of scientific publications and author organizations [16]. Since the 1980 s, the application of bibliometrics in medical research has expanded significantly, encompassing various disciplines such as obstetrics, gynecology, diabetic foot ulcers, radiology, and rheumatology [17–20]. Despite recent progress, no relevant bibliometric study exists on the TME-CRC relationship. In this paper, we searched all published relevant literature on inflammatory TME and CRC using the Web of Science Core Collection (WoSCC) database. We then visualized and analyzed the retrieved literature using Biblioshiny [21], VOSviewer [22], and CiteSpace [23], mapping collaborative networks and summarizing influential countries, institutions, authors, and journals. This comprehensive visualization offers insights into the field’s knowledge framework, research hotspots, and future trends. Our study, the first to use bibliometric tools to visualize the inflammatory TME-CRC relationship, contributes to CRC treatment strategies and improves patient prognosis, with implications for clinical translational medicine.

## Materials and methods

### Search strategy

**Fig. 1:**
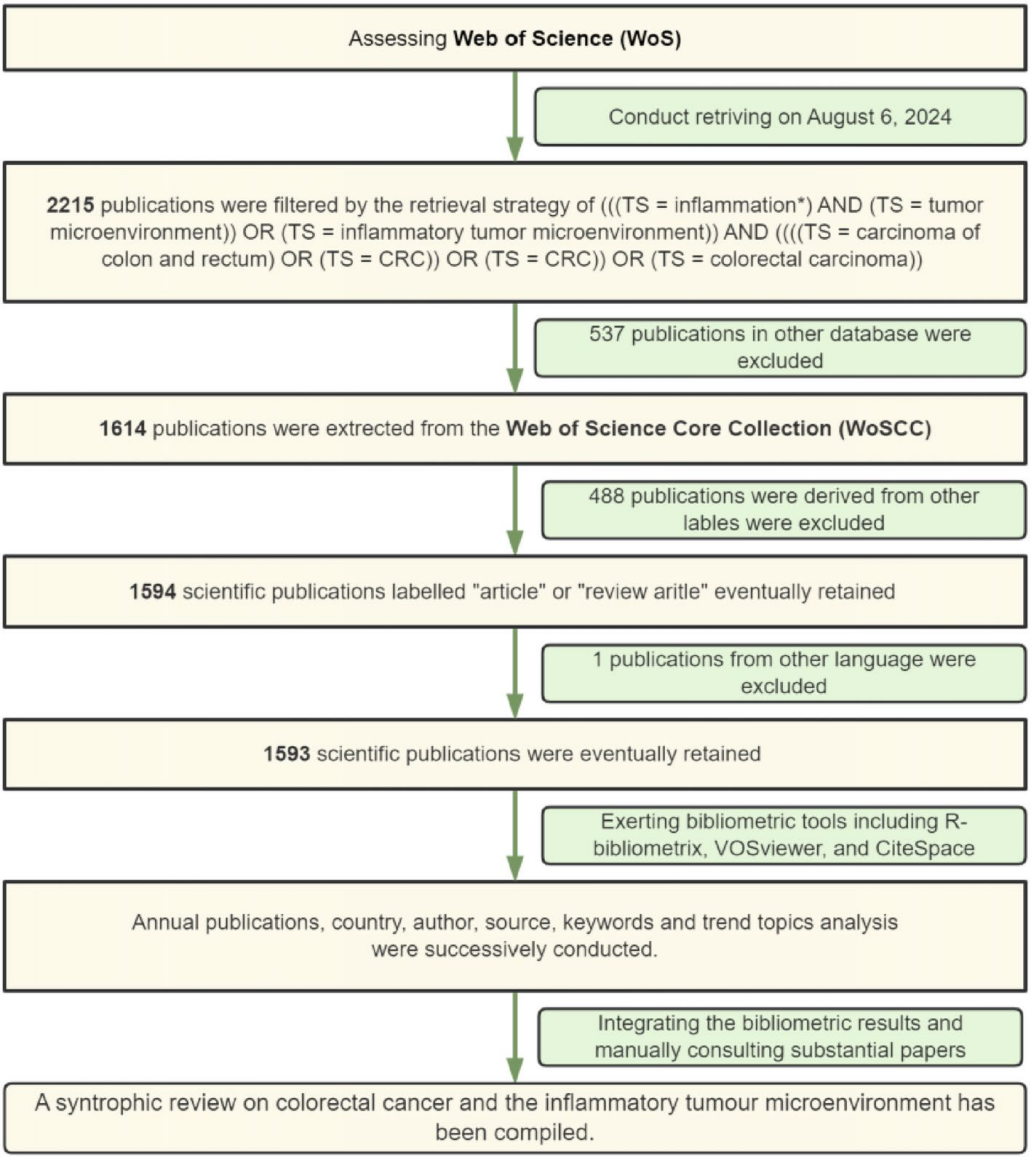
Integrated retrieval procedures and inclusion-exclusion criteria

The Science Citation Index, Expanded WoSCC database (https://www.webofscience.com/wos/woscc/basic-search), was conducted as the data source. The retrieval time was August 6th, 2024; the retrieval formula is (((TS = inflammation*) AND (TS = tumor microenvironment)) OR (TS = inflammatory tumor microenvironment)) AND ((((TS = carcinoma of colon and rectum) OR (TS = CRC)) OR (TS = colorectal carcinoma)) AND LA = (English). The literature type is “article” or “review article”; 488 literature with other lable were excluded (Detailed information can be found in Fig. 1). The time horizon is January 1 st, 2000, to August 6th, 2024. All 1593 literatures complete records and citation information were extracted and exported to TXT files as original records (Supplemental file 1). Then, it was imported into the metrology software for further translation. All retrieval and analysis processes are presented in a flowchart (Fig. 1).

### Data analysis

The R package “bibliometrix” (version 3.2.1) (https://www.bibliometrix.org), VOSviewer (version 1.6.18) [22] and CiteSpace (version 6.1.R1) [23] are commonly used bibliometric analysis software to extract critical information from numerous publications. This essential information is often used to build collaborations, co-citation, co-occurrence networks, etc., and generate visual maps. We first analyzed 1593 CRC and inflammatory TME-related studies for annual publications and their citations using the R package “bibliometrix”, the code during the current study is available in the Supplemental file 2. Subsequently, we visualized and examined the publication volume, influence, and collaborative network of countries, institutions, authors, and journals. At the same time, high-impact literature, keywords, etc., were used to build co-citation and co-occurrence networks to realize the depiction of the evolution of research hotspots and the historical trajectory of this field to construct a knowledge framework of CRC and inflammatory TME ultimately. On this basis, we also validated the above results using both VOSviewer (version 1.6.18) and CiteSpace (version 6.1.R1) to increase the credibility of the results.

## Results

### Temporal trends in publications of inflammatory TME in CRC research

The relevant literature published each year can reflect the size and trend of a particular research area. According to our search strategy, 1593 articles related to CRC inflammatory TME from 68 countries have been retrieved since the 21 st century, and the number of publications has generally shown an increasing trend since 2000, although accompanied by some fluctuations during this period. The overall trend in the number of publications can be roughly categorized into three time periods, with relevant publications in this field at a low level until 2012, when it was still in its infancy. After 2013, the number of publications showed an explosive growth trend and maintained a high growth level in the last five years. This may be attributed to the explosion and popularisation of high-throughput sequencing technologies such as sequencing and single-cell sequencing (Figure S1A). In addition, the number of annual citations as a whole has also maintained a rapid growth rate (Figure S1B). These findings may indicate that inflammatory TME’s role in CRC is gaining attention and has great potential for development in clinical and basic experiments.

### Most influential countries and institutions on the inflammatory TME in CRC research

**Fig. 2:**
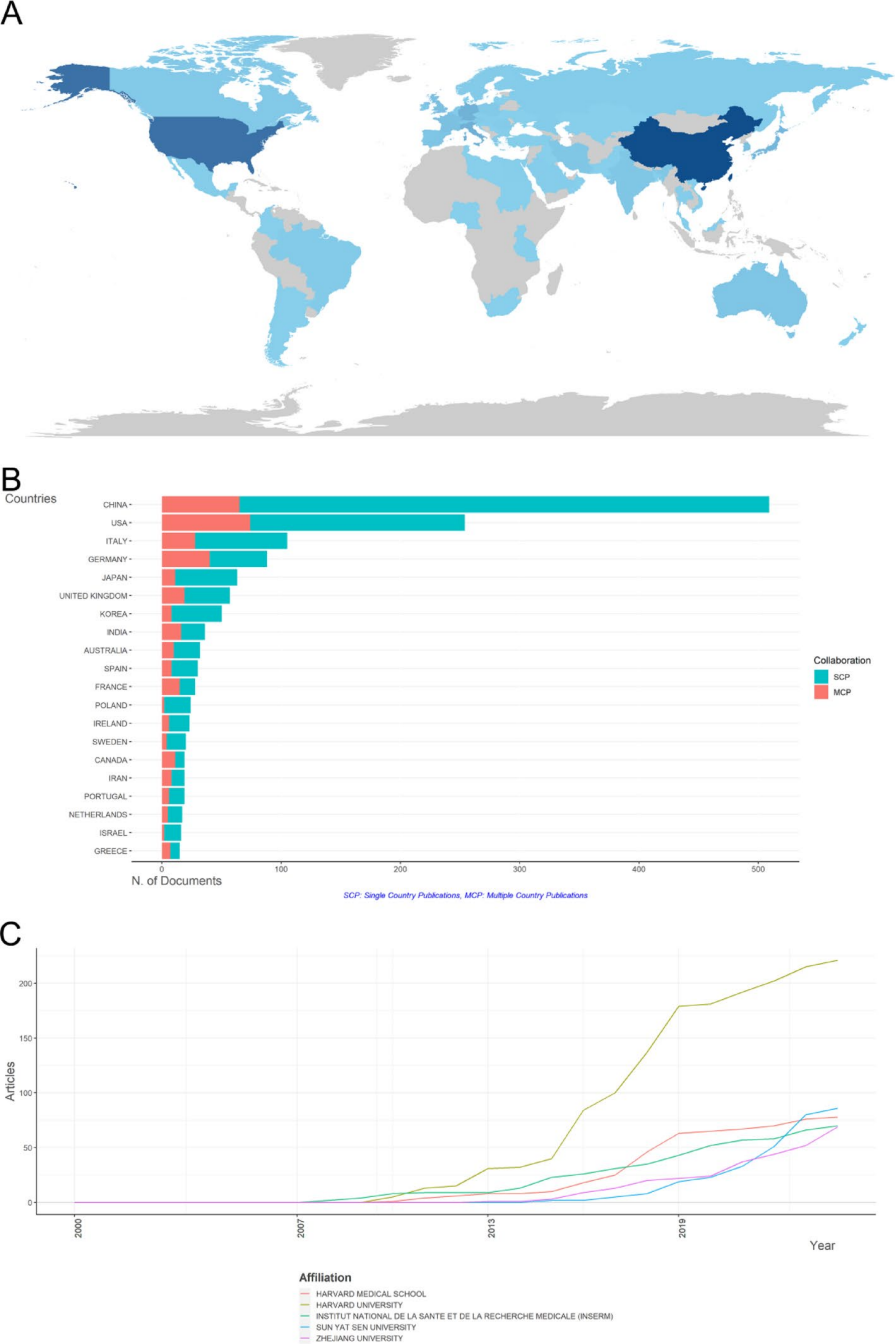
Countries/regions at the center of research related to the inflammatory TME with CRC. **A** The current state of research about inflammatory TME with CRC in each country worldwide, with darker colors indicating more research. **B** SCP and MCP production for the top 10 most productive countries/regions. **C** Top 10 institutions with the highest outputs in inflammatory microenvironment and TME research

The map of national publication outputs shows the distribution and number of publications in each country/region (Fig. 2A). China and the United States are prolific and influential in CRC and inflammatory TME research. Specifically, China has the most publications (*n* = 1808) with a total of 14,664 citations (Figure S2A), followed by the United States (1283 records cited 19618 times), Italy (400 records mentioned 13682 times) and Germany (388 records mentioned 6241 times). Notably, 70% of the top ten countries in the ranking hail from Europe, America, and Australia, while the remaining 30% originate from Asia. Notably, all of these countries, with the exception of China, are developed nations.

Figure S2B reveals a tight-knit collaboration among the primary research nations, underscoring the high level of international academic exchange and globalization within this field. Notably, the thickest link is observed between China and the United States, indicative of substantial mutual contributions and collaborations in research endeavors. We selects the top five countries in terms of the number of publications over time for the last 20 years (Figure S2C). The United States stands as the earliest pioneer in this research field, significantly outpacing other nations in both the inception year and the sheer volume of research outputs. Notably, China published its first study in this domain only in 2005, but within the subsequent decade, it has surpassed other countries to become the second most prolific nation, closely trailing the United States. Remarkably, China’s research output experienced an explosive growth after 2019, ultimately surpassing the United States in 2021 to emerge as the leading contributor of research publications in this field.

To visually analyze the output volume and collaborative relationships among nations in this field, we have generated a visual map of national collaborations. In this map, the size of a node represents the number of publications by a country, while the thickness of the connecting lines signifies the intensity of collaboration between nations. In addition, Table 1; Fig. 2B show the number of articles published by a single-country publications (SCP) and multicountry publications (MCP) for the 20 countries/regions with the highest productivity. The United States and China stands out as the country with the highest number of publications involving multi-national collaboration.

**Table 1:** The number of SCP and MCP for the 20 countries/regions with the highest productivity

Rank	Country	Publications	Proportion of Publications (%)	SCP	MCP	Proportion of MCP (%)
1	CHINA	316	33.10%	277	39	12.30%
2	USA	163	17.10%	102	61	37.40%
3	GERMANY	61	6.40%	32	29	47.50%
4	ITALY	47	4.90%	33	14	29.80%
5	JAPAN	44	4.60%	36	8	18.20%
6	UNITED KINGDOM	40	4.20%	26	14	35.00%
7	KOREA	34	3.60%	28	6	17.60%
8	SPAIN	18	1.90%	12	6	33.30%
9	IRELAND	17	1.80%	12	5	29.40%
10	SWEDEN	15	1.60%	12	3	20.00%
11	AUSTRALIA	14	1.50%	8	6	42.90%
12	FRANCE	13	1.40%	6	7	53.80%
13	CANADA	12	1.30%	5	7	58.30%
14	POLAND	12	1.30%	12	0	0.00%
15	SWITZERLAND	12	1.30%	4	8	66.70%
16	PORTUGAL	11	1.20%	6	5	45.50%
17	AUSTRIA	10	1.00%	4	6	60.00%
18	INDIA	9	0.90%	6	3	33.30%
19	ISRAEL	9	0.90%	7	2	22.20%
20	NETHERLANDS	9	0.90%	7	2	22.20%

*SCP* single-country publications, *MCP* multicountry publications

All institutions involved in CRC and inflammatory TME research were ranked according to the number of publications. Figure 2C shows the top 10 institutions, of which five were from China(50%), three were from United States (30%), one were from France (10%), one was from Portugal (10%). The top five most output institutions, which were Harvard University (*n* = 221), Sun Yat-Sen University (*n* = 86), Harvard Medical School (*n* = 78), Institut national de la santé et de la recherche médicale (*n* = 70), ZheJiang University (*n* = 69). Among the top ten institutions, 50% were from China, this may reaffirming China’s significant contribution and academic influence within this field. From the time dimension (Figure S2D), Harvard University remains at the forefront, with its research output exceeding other institutions.

### Most influential journals on the inflammatory TME in CRC research

**Fig. 3:**
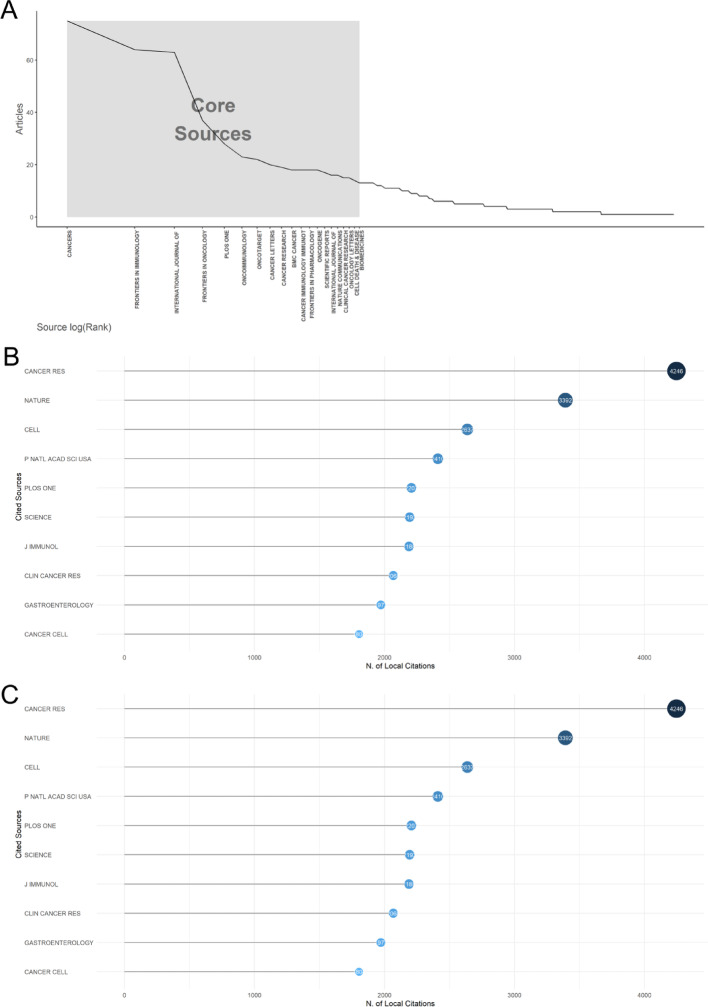
Critical sources to access the research frontiers and information on inflammatory TME with CRC research. **A** Core journals of inflammatory TME with CRC research based on Bradford’s Law. **B** The top 10 prolific journals on inflammatory TME with CRC research. **C** The 10 most cited journals in inflammatory TME with CRC research

Since 2000, 501 sources have published articles on CRC and inflammatory TME research. According to Bradford’s law, 20 high-production journals were categorized as core sources based on the number of publications (Fig. 3A). A journal’s impact is often gauged by the number of publications it hosts and the frequency of citations it receives. Hence, this study employs the Biblioshiny software to conduct a visual analysis of the top 10 journals with the highest number of publications in the field of inflammatory TME in CRC, subsequently calculating their average citation counts (see Table 2 for a detailed breakdown). The total number of articles published in the top 10 academic journals was 369 (Fig. 3B), totaling 14,545 citations (Fig. 3C). The academic journal *Cancers* published the highest number of articles (*n* = 75), which were cited 870 times; the remaining top five journals with the highest number of publications were *Frontiers in Immunology* (64 records cited 1,627 times), International Journal of Molecular Sciences (63 records cited 1155 times), and *Frontiers in Oncology* (37 records cited 216 times). *Cancer Research*, although not the most published (*n* = 19), was the most cited with 4,246 citations, followed by Nature (3,392 citations) and Cell (2,637 citations); these productive journals were essential sources of knowledge and had a significant impact on the perception of CRC and inflammatory TME research. Focusing on these journals will help provide faster access to cutting-edge research and critical information on the inflammatory TME in the CRC field.

**Table 2:** Top 10 sources with the highest productivity in the inflammatory TME with CRC research field

Sources	Articles	Citations	Averge citation per paper
Cancers	75	870	11.60
Froniers in Immunology	64	1627	25.42
International Journal of Molecular Science	63	1155	18.33
Frontiers in Oncology	37	645	17.43
Plos One	28	2207	78.82
Oncoimmunology	23	705	30.65
Oncotaget	22	1494	67.91
Cancer Letters	20	938	46.90
Cancer Research	19	4246	223.47
BMC Cancer	18	658	36.56
Cancer Immunology Immunotherapy	18	669	37.17

In addition, Figure S3 shows the growth in productivity over time for the top five most productive journals. Plos *One* was the first to publish research related to CRC and inflammatory TME. The other four significant journals started to publish related research articles one after the other after 2014, with *Cancers* appearing as the latest but the fastest growth in published articles. *Cancers* were the newest journal to emerge but are also the fastest-growing publication.

### Most influential authors on the inflammatory TME in CRC research

**Fig. 4:**
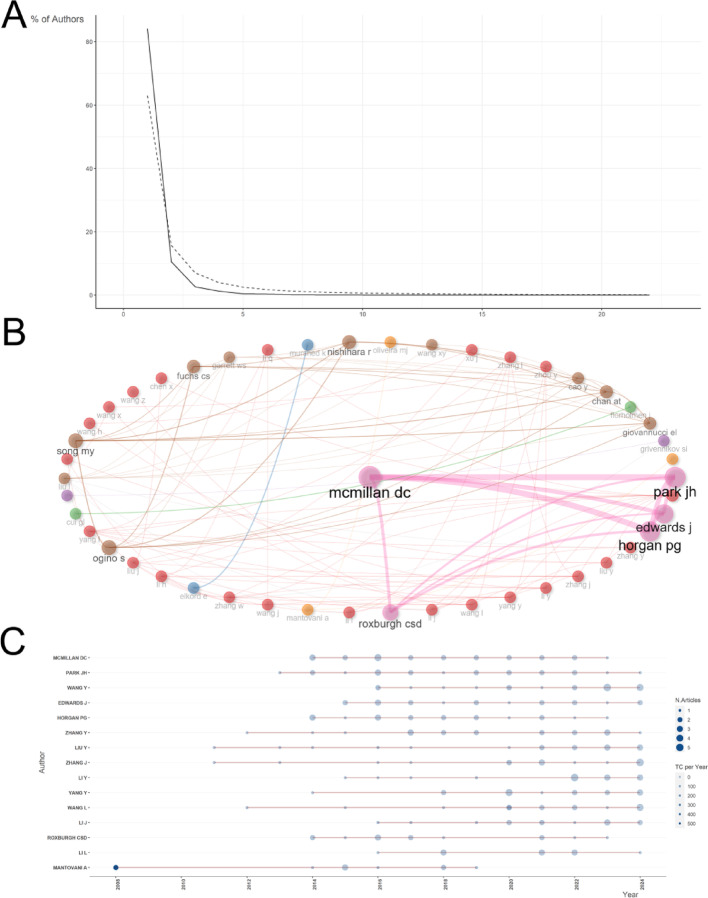
Primary authors of inflammatory TME with CRC research production and collaboration. **A** Lead authors of inflammatory TME with CRC studies based on Lotka’s law. **B** Most production authors’ collaboration network on inflammatory TME with CRC research. **C** The top 158 most productive authors on inflammatory TME with CRC research over time

Analyzing the number of publications by authors can provide insights into the representative scholars and core research capabilities within a particular field of study. Since 2000, more than 9786 authors have been involved in CRC and inflammatory TME research publications, and 17 have published more than 10 publications, and 84.2% of authors have issued one relevant publication (Fig. 4A). We identified the top 10 most productive authors, with 177 articles accounting for 11.11% of all articles. Figure S4A-C shows the top 10 most productive authors, the top 10 most cited authors, and the top 10 most locally influential authors as measured by the H-index. McMillan Dc (*n* = 22, 112 citations), Park Jh (*n* = 20, 93 citations), Wang Y (*n* = 19, mentioned 14 times), Edwards J (*n* = 18, cited 66 times) and Horgan pg (*n* = 18, 105 citations). Notably, Mantovani A, with only 11 publications, achieves the highest citation count of 200, ranking first and becoming the most cited author in this field. This underscores MANTOVANI A’s potential status as a leading figure, with each article contributing significantly to the profound impact of his research. In addition, according to the authors’ collaborative network diagram (Fig. 4B), Mcmillan DC, Horgan PG, and Park JH seem to be the authors with the essential collaborative networks. Figure 4C depicts the temporal publication trends of the top 15 authors, highlighting the evolution of their scholarly endeavors. Li Y and Li J are the authors who have been more active in the last two years.

### Most influential publications on the inflammatory TME in CRC research

**Fig. 5:**
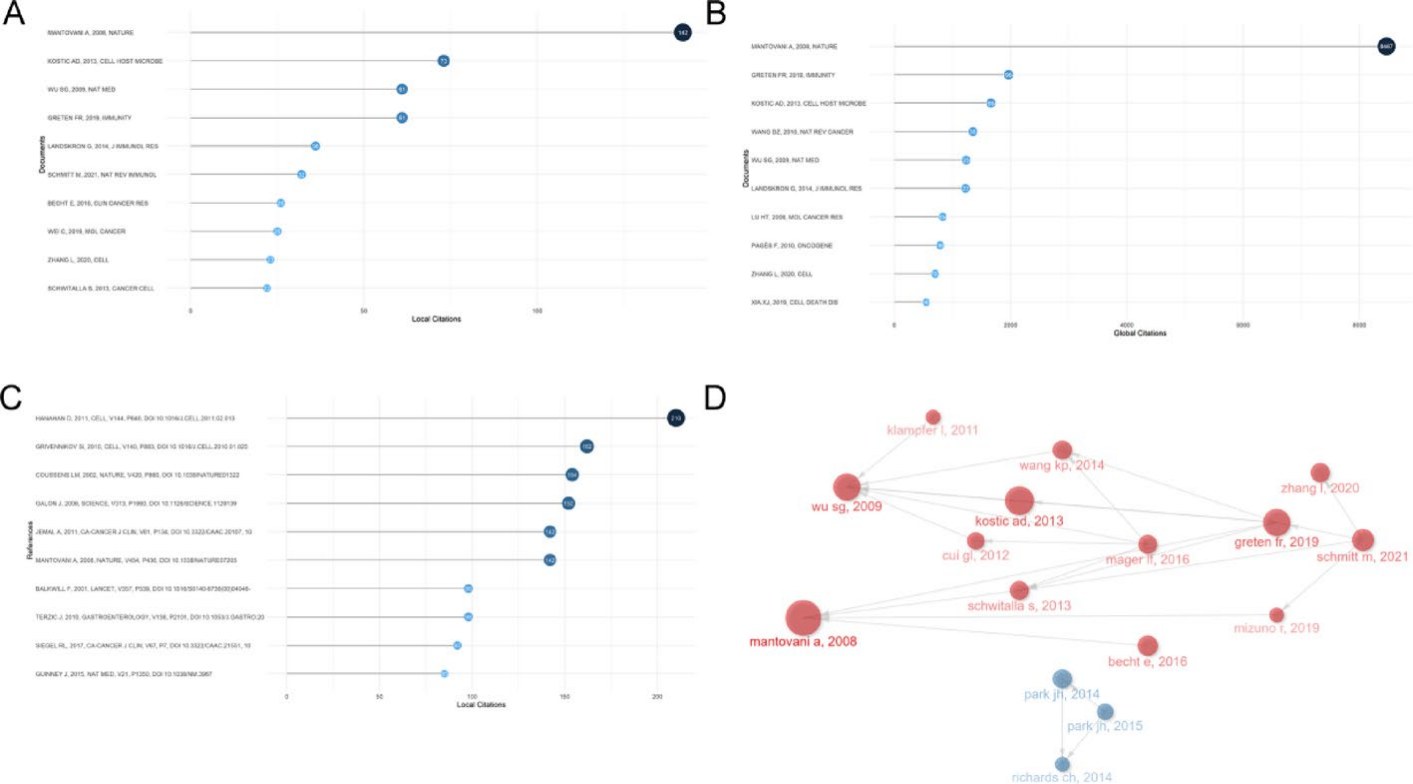
Global and local citation analyses of publications on inflammatory TME with CRC research have received more attention. **A** Top 10 most local cited publications on inflammatory TME with CRC research. **B** Top 10 most global cited publications on inflammatory TME with CRC research. **C** Top 10 most local cited references on inflammatory TME with CRC research. **D** Historical direct citation network. Each dot represents a document and is labeled with first author’s surname and the publication year

Cited literature provides directions for CRC research. Locally cited literature refers to citations within the search set, while globally cited literature appears in the Web of Science database. Highly globally cited literature has a significant impact on the database’s literature. Conversely, locally cited literature strongly influences CRC tumor-related microenvironment research. Table 3 shows the top 10 most locally cited documents, including their authors, key conclusion and year of publication, respectively. Among them are 4 documents with no less than 60 local citations, and the top-ranked document has been cited 142 times (Fig. 5A). In addition, there were 5 documents with more than 1,000 global citations, and the top-ranked document was mentioned 8,467 times (Fig. 5B). In addition, of the highly cited references, eight references had more than 50 local citations (Fig. 5C and Table 4).

**Table 3:** Top 15 most local cited publications on inflammatory TME with CRC research

Rank	Title	Journal	Author	Year	Local Citations	Global Citations	LC/GC Ratio (%)
1	A human colonic commensal promotes colon tumorigenesis via activation of T helper type 17 T cell responses [24]	NAT MED	Wu SG	2009	28	1163	2.41
2	Fusobacterium nucleatum potentiates intestinal tumorigenesis and modulates the tumor-immune microenvironment [25]	CELL HOST MICROBE	KOSTIC AD	2013	27	1511	1.79
3	The relationship between tumour stroma percentage, the tumour microenvironment and survival in patients with primary operable colorectal cancer [26]	ANN ONCOL	PARK JH	2014	17	134	12.69
4	Evaluation of a tumor microenvironment-based prognostic score in primary operable colorectal cancer [27]	CLIN CANCER RES	PARK JH	2015	15	52	28.85
5	The clinical utility of the local inflammatory response in colorectal cancer [28]	EUR J CANCER	RICHARDS CH	2014	14	74	18.92
6	Immune and Stromal Classification of Colorectal Cancer Is Associated with Molecular Subtypes and Relevant for Precision Immunotherapy [29]	CLIN CANCER RES	BECHT E	2016	13	366	3.55
7	Interleukin-17 receptor a signaling in transformed enterocytes promotes early colorectal tumorigenesis [30]	IMMUNITY	WANG KP	2014	12	226	5.31
8	[31] Mismatch repair status in patients with primary operable colorectal cancer: associations with the local and systemic tumour environment [32]	BRIT J CANCER	PARK JH	2016	10	57	17.54
9	Single-Cell Analyses Inform Mechanisms of Myeloid-Targeted Therapies in Colon Cancer [33]	CELL	ZHANG L	2020	10	504	1.98
10	Innate immune signaling by Toll-like [34]	INFLAMM BOWEL DIS	FUKATA M	2009	9	119	7.56
11	Loss of p53 in enterocytes generates an inflammatory microenvironment enabling invasion and lymph node metastasis of carcinogen-induced colorectal tumors [35]	CANCER CELL	SCHWITALLA S	2013	9	211	4.27
12	Crosstalk between cancer cells and tumor associated macrophages is required for mesenchymal circulating tumor cell-mediated colorectal cancer metastasis [36]	MOL CANCER	WEI C	2019	9	373	2.41
13	Macrophage-derived IL-1beta stimulates Wnt signaling and growth of colon cancer cells: a crosstalk interrupted by vitamin D3 [37]	Oncogene	KALER P	2009	8	198	4.04
14	IL-33 promotes growth and liver metastasis of colorectal cancer in mice by remodeling the tumor microenvironment and inducing angiogenesis [38]	MOL CARCINOGEN	ZHANG Y	2017	8	99	8.08
15	The relationship between right-sided tumour location, tumour microenvironment, systemic inflammation, adjuvant therapy and survival in patients undergoing surgery for colon and rectal cancer [39]	BRIT J CANCER	PATEL M	2018	8	45	17.78

*LC* local citations,* GC* global citations

**Table 4:** Top 15 most local cited references on inflammatory TME with CRC research

Rank	Title	Journal	Author	Year	Citations
1	Hallmarks of cancer: the next generation	CELL	HANAHAN D	2011	105
2	Immunity, inflammation, and cancer	CELL	GRIVENNIKOV SI	2010	97
3	Type, density, and location of immune cells within human colorectal tumors predict clinical outcome	SCIENCE	GALON J	2006	94
4	Inflammation and cancer	NATURE	COUSSENS LM	2002	93
5	Global cancer statistics, 2012	CA-CANCER J CLIN	JEMAL A	2011	84
6	Inflammation and colon cancer	GASTROENTEROLOGY	TERZIC J	2010	71
7	Cancer-related inflammation	NATURE	MANTOVANI A	2008	69
8	Cancer statistics, 2009	CA-CANCER J CLIN	JEMAL A	2009	60
9	The consensus molecular subtypes of colorectal cancer	NAT MED	GUINNEY J	2015	49
10	IL-6 and Stat3 are required for survival of intestinal epithelial cells and development of colitis-associated cancer	CANCER CELL	GRIVENNIKOV S	2009	48
11	PD-1 Blockade in Tumors with Mismatch-Repair Deficiency	NEW ENGL J MED	LE DT	2015	48
12	IKKbeta links inflammation and tumorigenesis in a mouse model of colitis-associated cancer	CELL	GRETEN FR	2004	44
13	Inflammation and cancer: back to Virchow?	LANCET	BALKWILL F	2001	41
14	Macrophage diversity enhances tumor progression and metastasis	CELL	QIAN BZ	2010	39
15	Gene set enrichment analysis: a knowledge-based approach for interpreting genome-wide expression profiles	P NATL ACAD SCI USA	SUBRAMANIAN A	2005	36

The review article by Alberto Mantovani [40] with titled “Cancer-related inflammation” which was published in 2008 in *Nature*, was the most locally cited article (142 local citations and 8467 global citations). It was the most locally citated and globally citated documents, offer insights into the meticulous examination of cancer-linked inflammatory pathways unveils novel therapeutic targets, enhancing clinical diagnosis and refining tumor treatment strategies, as well as the potential mechanism for colorectal tumorigenesis and progression via a proinflammatory microenvironment. Crucially, within the historical direct citation network graph (Fig. 5D; Table 4), this article stands at the forefront in terms of both publication time and citation frequency, pioneering the developmental trajectory of numerous subsequent works. Followed by the article titled “Fusobacterium nucleatum potentiates intestinal tumorigenesis and modulates the tumor-immune microenvironment” by Aleksandar D. Kostic [25] in 2013 from Cell Host & Microbe (IF = 30.3) with 73 local citations and 1664 global citations. The article by Shaoguang Wu [24] with titled “A human colonic commensal promotes colon tumorigenesis via activation of T helper type 17 T cell responses.” which was published in 2009 in Nature Medicine (IF = 82.9), was the most locally cited article (28 local citations).

### Analysis of high-frequency keywords and three research hotspots based on the keyword co-occurrence

**Fig. 6:**
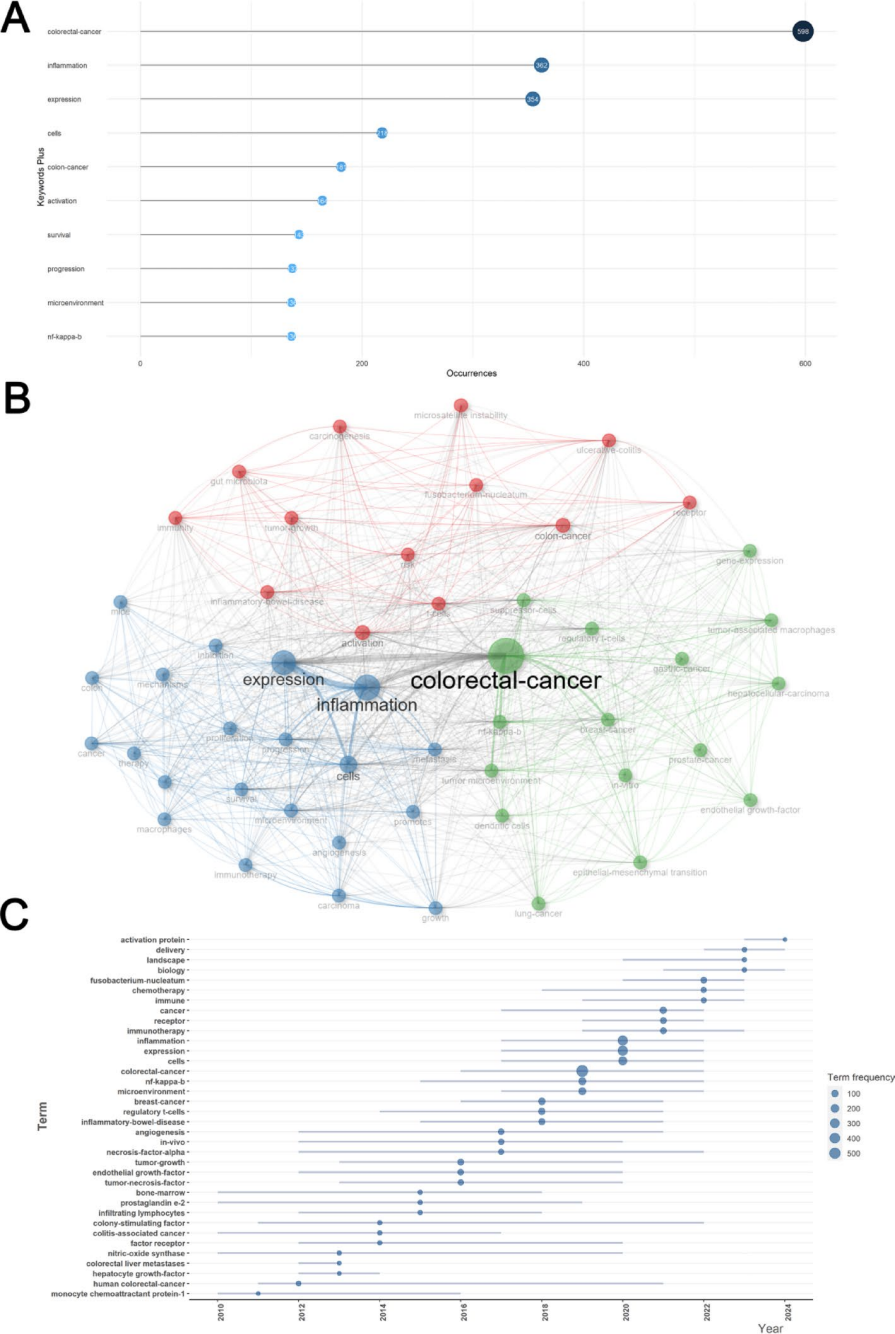
Analysis of most high-frequency keywords and three research directions given the keywords co-occurrence network. **A** Top 15 Most Frequent Keywords in inflammatory TME with CRC research. **B** Visualized keywords co-occurrence network for inflammatory TME with CRC research. These clusters mirror the initial research content and core area of research represented by the keywords. Each node represents a keyword, and the node size represents the occurrence frequency. The lines between keywords represent that they have co-occurred in the literature, and the thickness of the lines represents the frequency of co-occurrence. The three clusters were red, green, and blue [3]. Sketching historical trajectories and revealing research frontiers of inflammatory TME with CRC. *TME* tumor microenvironment,* CRC* Colorectal cancer

Using a tree diagram, we identified the top 50 high-frequency keywords for studies related to CRC and inflammatory TME. Figure 6A shows the top 10 keywords with the highest frequency of occurrence. Specifically, “colorectal cancer” had the highest frequency of occurrence (*n* = 598), followed by “inflammation” (*n* = 362), “expression” (*n* = 354), “cells” (*n* = 218), “colon-cancer” (*n* = 181), “activation” (*n* = 164),"survival” (*n* = 143), “progression” (*n* = 137), “microenvironment” (*n* = 136), and “nf-kappa-b” (*n* = 136). Next, we used Biblioshiny software to perform keyword co-occurrence analysis to form a keyword clustering network diagram and finally classified the related keywords into 3 clusters (Fig. 6B). In the keyword co-occurrence network graph, each node represents a keyword, and the node’s size represents its frequency of occurrence; the line between the nodes indicates the affinity between the keywords. To improve the accuracy, remove isolated nodes.

Cluster 1 (blue): Reveals IBD-associated immune dysregulation drives CRC carcinogenesis. critical keywords in this one group were “colon-cancer” (avg. per year 12.26 occurrences, *n* = 181), “activation” (avg. pub. per year 5.39 occurrences, *n* = 164), “t-cells” (avg. pub. per year 3.48 occurrences, *n* = 132) and “inflammatory-bowel-disease” (avg. pub. per year 5.39 occurrences, *n* = 80).

Cluster 2 (red): The effect of NF-κB-mediated TME remodeling in CRC progression. Critical keywords in this one group were “colorectal-cancer” (avg per year 13.61 occurrences, *n* = 598), “nf-kappa-b” (avg. pub. per year 2.52 occurrences, *n* = 136), “tumor microenvironment” (avg. pub. per year 2.83 occurrences, *n* = 65), and “breast-cancer” (avg. pub. per year 1.96 occurrences, *n* = 103).

Cluster 3 (green): Impact of CRC patient stratification and precision therapeutics. Critical keywords in this one group were “inflammation” (avg. pub. per year 4.78 occurrences, *n* = 362), “expression” (avg per year 11.43 occurrences, *n* = 354), “cells” (avg. pub. per year 7.17 occurrences, *n* = 218) and “survival” (avg. pub. per year 5.09 occurrences, *n* = 143).

### The research status and evolution trajectory on inflammatory TME in CRC

Using Biblioshiny software, a timeline visualization map (Fig. 6C) was constructed, reflecting the evolutionary trends. A minimum frequency of 5 was set for keywords, limiting yearly representatives to 3. Figure 6C visually presents, through a dot plot, the variations in keyword frequency within the research domain of inflammatory TME in TME from 2000 to 2024. The horizontal axis represents the years, while the vertical axis enumerates a series of pivotal research themes. Prior to 2013, CRC-related inflammatory TME attracted fewer prominent keywords, echoing trends in publication numbers. Topics then included “monocyte chemoattractant protein-1”, “nitric-oxide synthase”, “colorectal liver metastases”, and “hepatocyte growth-factor”. Scholars explored pathophysiological mechanisms in CRC’s inflammatory TME, still in its infancy. However, following 2016, the widespread of high-throughput sequencing technologies, including second-generation sequencing and single-cell sequencing, provided a catalyst for rapid development in this emerging field [41, 42]. This technological revolution catalyzed exponential growth in publication output, keyword frequency, and thematic diversity. Dominant keywords clustered around “colorectal cancer,” “inflammation,” and “expression.” Advanced bioinformatic approaches enabled mechanistic dissection of inflammatory TME’s cellular and molecular roles in cancer progression/metastasis, alongside the development of targeted therapeutic strategies.

Recent years have witnessed a paradigm shift toward clinical translation. Emerging keywords include “activating protein,” “delivery,” and “biology.” A notable frontier involves nanodelivery systems, such as pH-sensitive polymeric nanoparticles, achieving targeted chemotherapeutic release within TME to suppress TME growth/metastasis. This innovation exemplifies the field’s trajectory toward therapeutic application, positioning TME-targeted nanomedicine as a potential research hotspot [43].

## Discussion

### General information

The current study utilizes information visualization to delve into the literature on CRC and its inflammatory TME. From January 1, 2000, to August 6th, 2024, 1593 relevant articles were retrieved after careful screening. This growth trend indicates increasing researcher attention and lays a foundation for future CRC research. China, the US, Italy and Germany are the leading contributors in CRC inflammatory TME research. Harvard University tops the list with the most publications. In terms of collaboration, the US and China work closely together and engage with other countries. The academic journal *Cancers* has published the most significant number of articles and is the most popular journal studying inflammatory TME in CRC. The rest, in descending order, are *Frontiers in Immunology* and *Plos One.* Several authors, Mcmillan DC Park JH and Mantovani A are relative leaders in publications, citations, and impact index. Their research in this field is more durable and continuous regarding the time dimension.

### Countries and institutions productivity and collaboration

In terms of national and institutional output as well as international collaboration, China and the United States lead in production, followed by Germany and Italy. Most of the top ten countries are located in Europe, America, and Australia, indicating a strong English research axis in the field of CRC inflammatory TME. The geographic concentration of high-impact research outputs correlates systematically with national R&D investment levels, availability of advanced technological infrastructure (e.g., genomic/proteomic platforms), and density of elite research institutions. Notably, all countries actively contributing to this field except China rank among the top 15% globally for gross domestic expenditure on R&D (GERD)-to-GDP ratios. This pattern suggests that economic resources may influence research advancement in this domain. China’s rapid ascent aligns with strategic initiatives like the “Double First-Class” university program and National Key R&D Program funding, though its average citation rate per paper remains 18% below the field benchmark. This discrepancy indicates a research ecosystem still maturing in quality metrics and international collaboration networks. Collaboration between China and the United States is particularly tight, and such cooperation is crucial for addressing challenges global CRC inflammatory TME and setting the international research agenda.

### Author analysis

The distribution of authors reveals the knowledge structure and depth of the research field of inflammatory TME in CRC. The foundational figures in this domain are McMillan Dc, Park Jh, and Mantovani A. McMillan Dc and Park Jh are renowned for their extensive research and widespread collaborations in this area, having published the highest number of articles. Their research spans multiple aspects of CRC, particularly achieving notable results in studying the relationship between inflammatory TME and CRC prognosis. These studies have preliminarily elucidated the potential mechanisms underlying inflammatory cell involvement in colorectal CRC development and have offered theoretical insights for the exploration of therapeutic strategies in CRC. Notably, Professor Mantovani A, a prominent researcher in the field of inflammation and carcinogenesis, has published several highly cited articles [44, 45]. His research trajectory has introduced novel research perspectives and directions, which may contribute to advancing clinical translation, fostering academic discourse, and promoting collaborative efforts within this specialized domain.

### Sources analysis

The academic landscape of CRC research, particularly concerning the inflammatory TME, is shaped by key journals that serve as both disseminators of knowledge and indicators of scientific rigor. The academic journals Cancers, Frontiers in Immunology, and PLOS ONE have published the most significant number of articles and are the most popular for studying inflammatory TME in CRC. Such as in Frontiers in Immunology the study linking SPP1 + macrophages and fibroblasts to immunosuppressive networks in CRC liver metastases. This exemplifies its niche in pioneering mechanistic insights. PLOS ONE emphasises reproducibility through detailed methodology, consistent with the emphasis on data transparency in Nature’s sub-journals. Journals like Frontiers in Immunology facilitate cross-disciplinary dialogue, as seen in studies decoding TME immune evasion mechanisms. They are not only major disseminators of research findings but also indicators of research quality and relevance. Cancer Research has the highest number of citations despite not leading in the number of publications, Studies in Cancer Research on CRC and the inflammatory microenvironment focus on elucidating molecular mechanisms (e.g., NF-κB, IL-6/STAT3 pathways), cell interaction networks (e.g., TAMs, iCAFs), and clinical translation strategies (e.g., targeting CXCL12/CXCR4). These studies provide a critical theoretical foundation for understanding inflammation-driven CRC progression and offer new targets for developing precision treatment strategies. This journal may serve as a critical platform for pioneering work in the CRC inflammatory TME domain, particularly for studies with transformative potential. These journals cover various aspects of CRC research, including basic research, clinical research, and translational medicine, fostering interdisciplinary integration with immunology research and providing a broad platform for academic exchange among researchers.

### Research hotspot analysis

Keyword analysis can help us accurately grasp the historical evolutionary trajectory and research frontiers of research hotspots within the research field related to CRC inflammatory TME. The ten most frequently occurring keywords were “colorectal cancer”, “inflammation”, “expression”, “cells”, “colon cancer”, “activation”, “survival”, “progression”, “microenvironment” and “nf-kappa-b”. On this basis, high-frequency keywords were analyzed using Biblioshiny software, and a keyword co-occurrence network was constructed to obtain three keyword clusters. The following three current research directions were involved: immunomodulatory mechanisms in IBD-associated carcinogenesis, NF-κB-mediated TME remodeling in tumor progression, and CRC patient stratification and precision therapeutics.

### Research theme evolution: hot knowledge spectrum and future trends

Through trend topic analysis, we systematically traced the thematic evolution of inflammatory TME research in TME, with its core trajectory divided into three stages:

#### Theoretical foundations and mechanistic exploration (2000–2013)

Early investigations centered on CRC tumor biology, encompassing genetic alterations and the emerging concept of the TME. Paget’s “seed and soil” hypothesis and Hanahan’s hallmarks of cancer theory [11] established the conceptual framework for TME research. Hanahan et al. delineated six cancer hallmarks: self-sufficiency in growth signals, insensitivity to antigrowth signals, evasion of apoptosis, limitless replicative potential, sustained angiogenesis, and tissue invasion/metastasis. Notably, two of these hallmarks—sustained angiogenesis and tissue invasion/metastasis—were directly associated with the pro-tumorigenic roles of TME components. The NF-κB pathway emerged as a frequently cited keyword (*n* = 136 occurrences) in this context, with experimental validation demonstrating its regulatory role in inflammatory cytokine networks (e.g., IL-6, TNF-α) that drive tumor cell proliferation and angiogenesis [46–48]. Collectively, these hallmarks provided theoretical foundations for subsequent mechanistic studies on cancer initiation and progression, forming the basis for translational research in TME modulation.

#### Technological advancements and hotspot expansion (2014–2020)

The widespread adoption of high-throughput sequencing technologies, has significantly enhanced molecular characterization of TME. A landmark study identified exhausted CD8 + T cell subsets in CRC TME through single-cell RNA sequencing, with PD-1 expression demonstrating negative correlation to patient survival [49, 50]. Immunotherapy emerged as a focal point during this period (high-frequency keyword, *n* = 120 occurrences), with gut microbiota alterations influencing glycerophospholipid metabolism and potentially modulating immune-related cytokines (IFN-γ, IL-2) to enhance PD-1 inhibitor efficacy in MSS-CRC [31]. The impact of immunotherapy and molecular targeted therapy on tumor progression became a research hotspot, driven by single-cell sequencing’s ability to resolve tumor heterogeneity.

#### Clinical translation and multidisciplinary integration (2021–2024)

Artificial intelligence (AI) applications in oncology have gained prominence. Deep learning-based CT image analysis models achieved precisely predict immunotherapy response by quantifying TME spatial heterogeneity [51]. This approach enabled simultaneous assessment of immune and stromal TME status alongside radiological prognostic features. AI-assisted colonoscopy demonstrated higher adenoma detection rates than conventional methods [52]. Additionally, nanodelivery systems such as pH-responsive polymeric nanoparticles achieved targeted release of chemotherapeutic agents within TME, showing significently enhanced tumor suppression compared to conventional delivery in animal models [53].

### Future directions and technological synergies

*Technical Integration Directions* In the realm of technical integration, multi-omics data integration will be employed to construct TME atlases by combining metabolomic, proteomic, and spatial transcriptomic datasets [54, 55]. AI-assisted target prediction platforms will be developed, utilizing machine learning algorithms to uncover associations between exosomal proteins and immune evasion mechanisms [56].

*Clinical Translation Directions* For personalized immunotherapy, patient stratification based on TME immune phenotypes (e.g., IM-type/inflammatory phenotypes) will be implemented to identify beneficiaries. Therapeutic strategies will focus on modulating the microbiota-metabolism-immune axis through targeted probiotic formulations targeting specific bacterial genera. In interdisciplinary collaboration, medical-engineering partnerships will develop TME-responsive drug delivery systems such as pH-sensitive nanoparticles [56–58].

*Knowledge Trajectory Summary* Since 2000, research has progressed along trajectories moving from local to systemic, mechanistic to clinical, and monodisciplinary to interdisciplinary. The scope expanded from individual TME components (e.g., CAFs, inflammatory cytokines) to systemic inflammation and gut microbiota regulation. Early mechanistic focus on molecular pathways (e.g., EMT, signaling cascades) has shifted toward clinical translation of immunotherapy (e.g., immune sensitization strategies for MSS-CRC). Integrative approaches combining metabolomics (exosomal proteomics), microbiology (gut microbiota), and AI (biomarker prediction) are driving precision medicine advancements.

In conclusion, research in the TME inflammatory TME field has achieved a paradigm shift from descriptive to mechanistic and now to intervention-oriented studies. Cross-disciplinary studies on inflammatory signaling pathways (e.g., TNF, NF-κB) and immunotherapy (e.g., PD-1 inhibitors) represent current research hotspots. Future efforts should prioritize technology-driven, clinically oriented integration of multi-omics platforms to deepen understanding of tumor-immune-microbiome interactions, ultimately realizing precision medicine goals.

*Advantages and Shortcomings* Our study offers conducted the first comprehensive and objective bibliometric analysis of relevant studies. This study employed bibliometric analysis to systematically map the evolution of research hotspots in the field of inflammatory TME and CRC. Key signaling pathways (e.g., NF-κB) and emerging intervention strategies (e.g., immunotherapy) were identified through temporal analysis of publication trends. The findings predict future research directions and providing a potiential evidence-based guidance for precision medicine approaches in CRC management. While this study systematically mapped the intellectual landscape of the inflammatory TME in TME via bibliometric analysis, several limitations persist. First, data were sourced primarily from the Web of Science Core Collection, which may omit pivotal studies due to database coverage gaps and keyword selection biases. Second, bibliometric methods rely on historical data, delaying the capture of emergent frontiers. Third, algorithm variations in visualization tools influence network layouts and clustering, as seminal/highly cited works dominate co-citation networks, obscuring under-cited but emerging studies. Finally, despite efforts to maintain objectivity, contextual limitations may bias interpretations of certain conclusions.

## Conclusion

In the current study, the relationship between inflammatory TME and the development of CRC was comprehensively assessed from a bibliometric perspective using various authoritative metrological tools for mutual validation. Research in this field has shown an upward trend since 2000, especially since the explosion of high-throughput sequencing technology. The countries, institutions, journals, and authors that contributed most to the field were the United States and China, *Cancers* and *Frontiers in Immunology*, Mcmillan DC, Park JH and Mantovani A. Combining multiple visualization and analysis methods and reviewing a large amount of literature, we have discovered that Immunomodulatory Mechanisms in IBD-Associated Carcinogenesis, NF-κB-Mediated TME Remodeling in Tumor Progression, and CRC patient stratification and precision therapeutics may three critical hot topics in inflammatory TME in CRC research.

## Supplementary Information

Supplementary Material 1

## Data Availability

The original contributions presented in the study are included in the article/Supplementary Material, further inquiries can be directed to the corresponding authors.

## Code availability

The code during the current study are available in the supplementary materials.
