# Supplementary material for: Mapping the global intellectual landscape of inflammatory tumor microenvironment in colorectal cancer pathogenesis and prognostic research since this century
Source: Discov Oncol. 2025 Oct 14;16:1884. doi: 10.1007/s12672-025-03524-w (PMC12521722; doi:10.1007/s12672-025-03524-w)
Supplement: Supplementary file 1 — Supplementary Material 1 [file 12672_2025_3524_MOESM1_ESM.zip › supplementary materias/Supplemental Figures.docx]

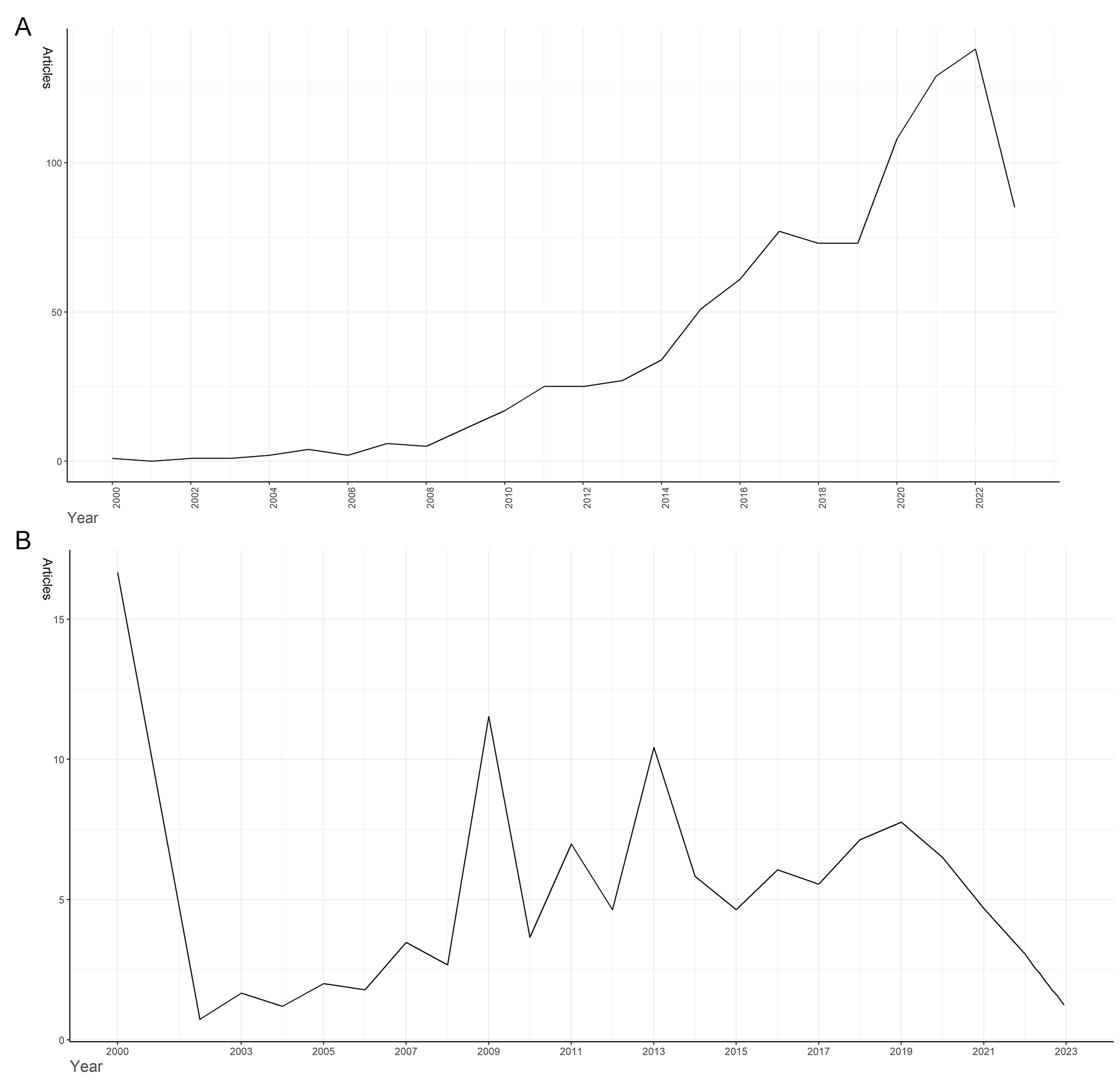


**Figure S1.** The growth of inflammatory TME with CRC has been steadily increasing and arousing increasing concern recently.

(A) The growth of inflammatory TME with CRC research was steadily increasing and arousing increasing concern from 2000 to 2024.

(B) The citation growth of inflammatory TME with CRC research gradually increased, fluctuating from 2000 to 2024.


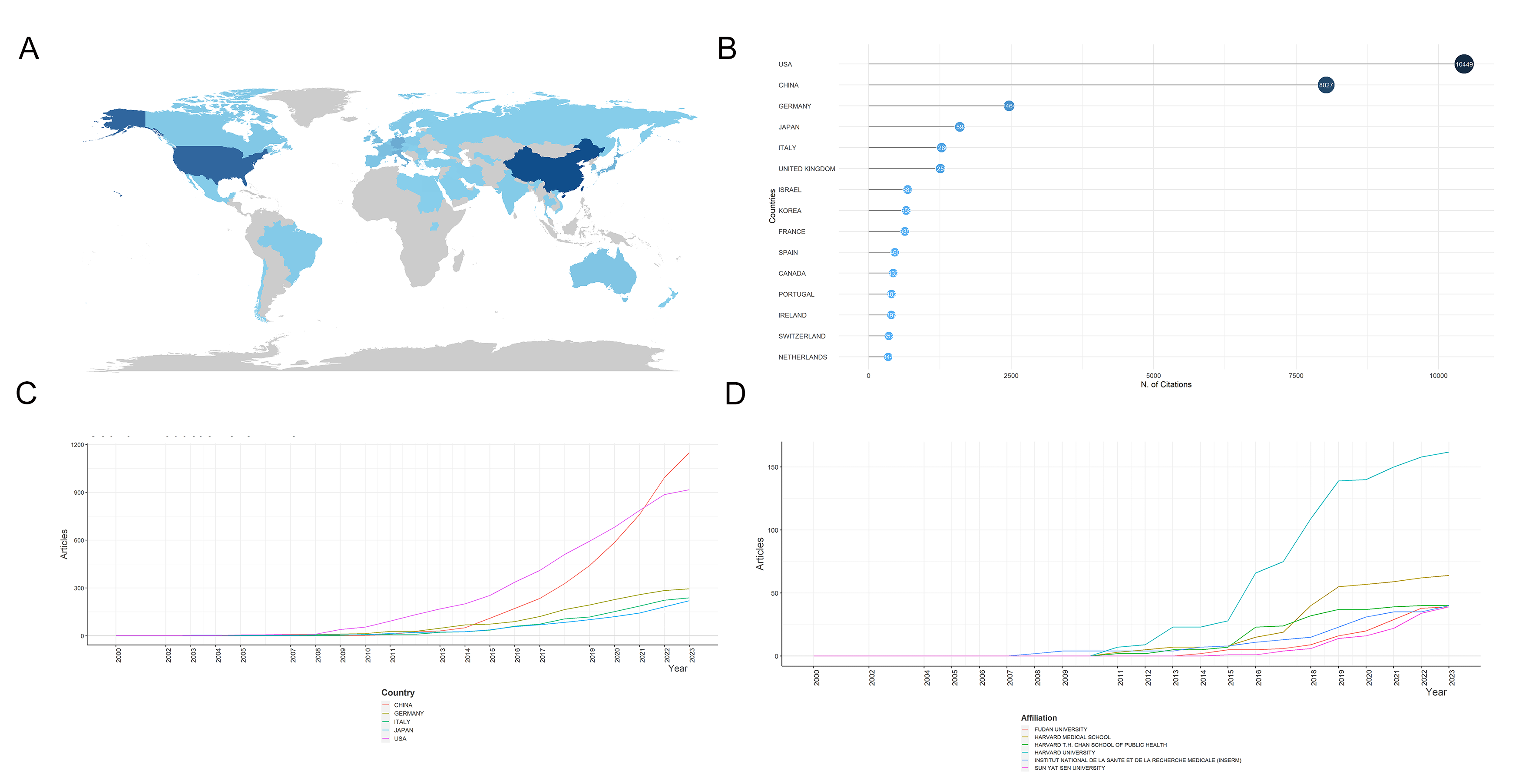


**Figure S2.** Countries/Regions production distribution world map of inflammatory TME with CRC research.

1. World map of national/regional outputs and collaborations in inflammatory TME and CRC research.
2. Top 10 countries/regions with the highest outputs in inflammatory microenvironment and colorectal cancer research
3. Top countries leading research on inflammatory TME with CRC research.
4. Top global institutions with the most research outputs on inflammatory TME with CRC research.


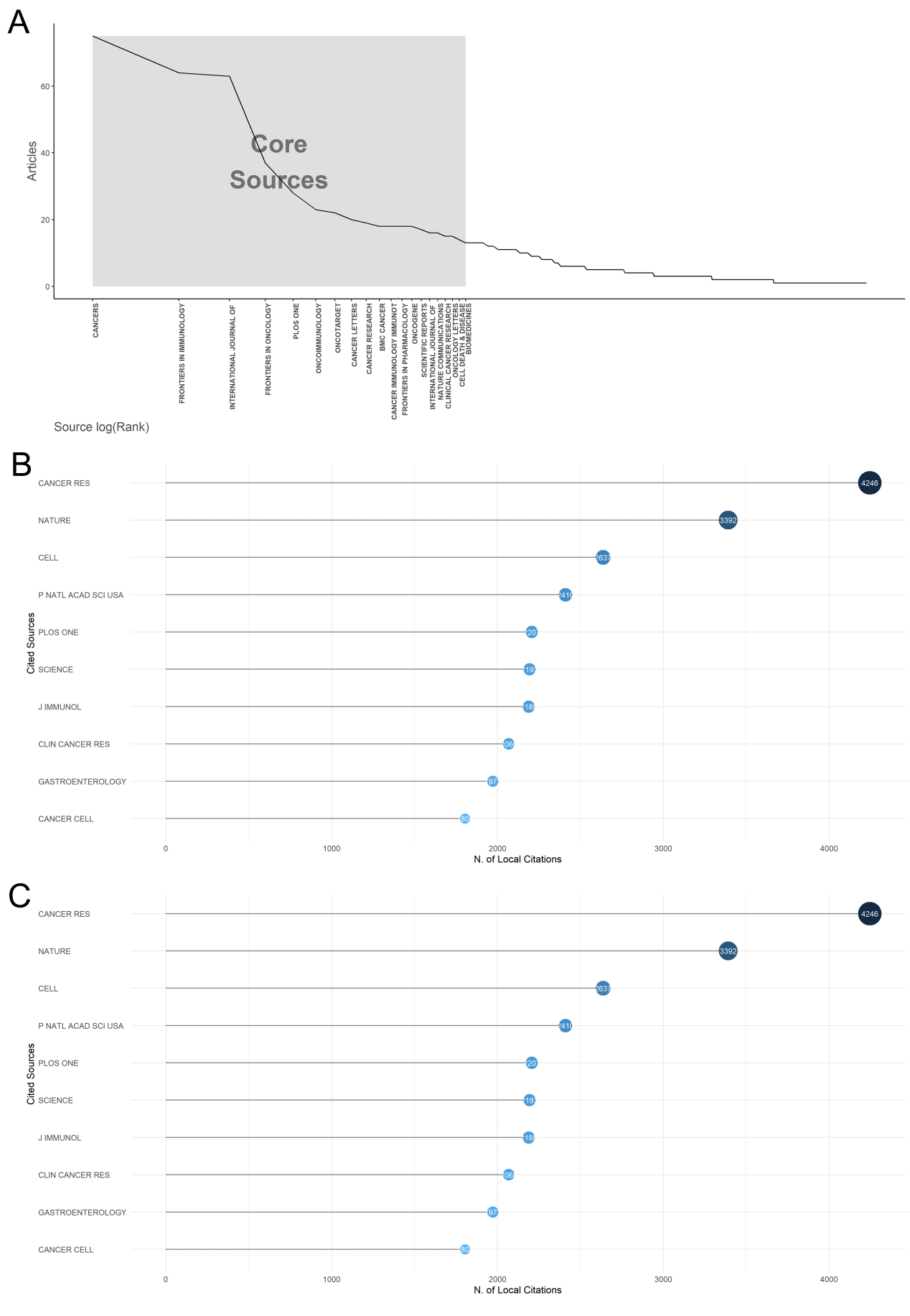


**Figure S3.** Critical sources to access the research frontiers and information on inflammatory TME with CRC research.

(A) Core journals of inflammatory TME with CRC research based on Bradford's Law.

(B) The top 10 prolific journals on inflammatory TME with CRC research.

(C) The 10 most cited journals in inflammatory TME with CRC research.


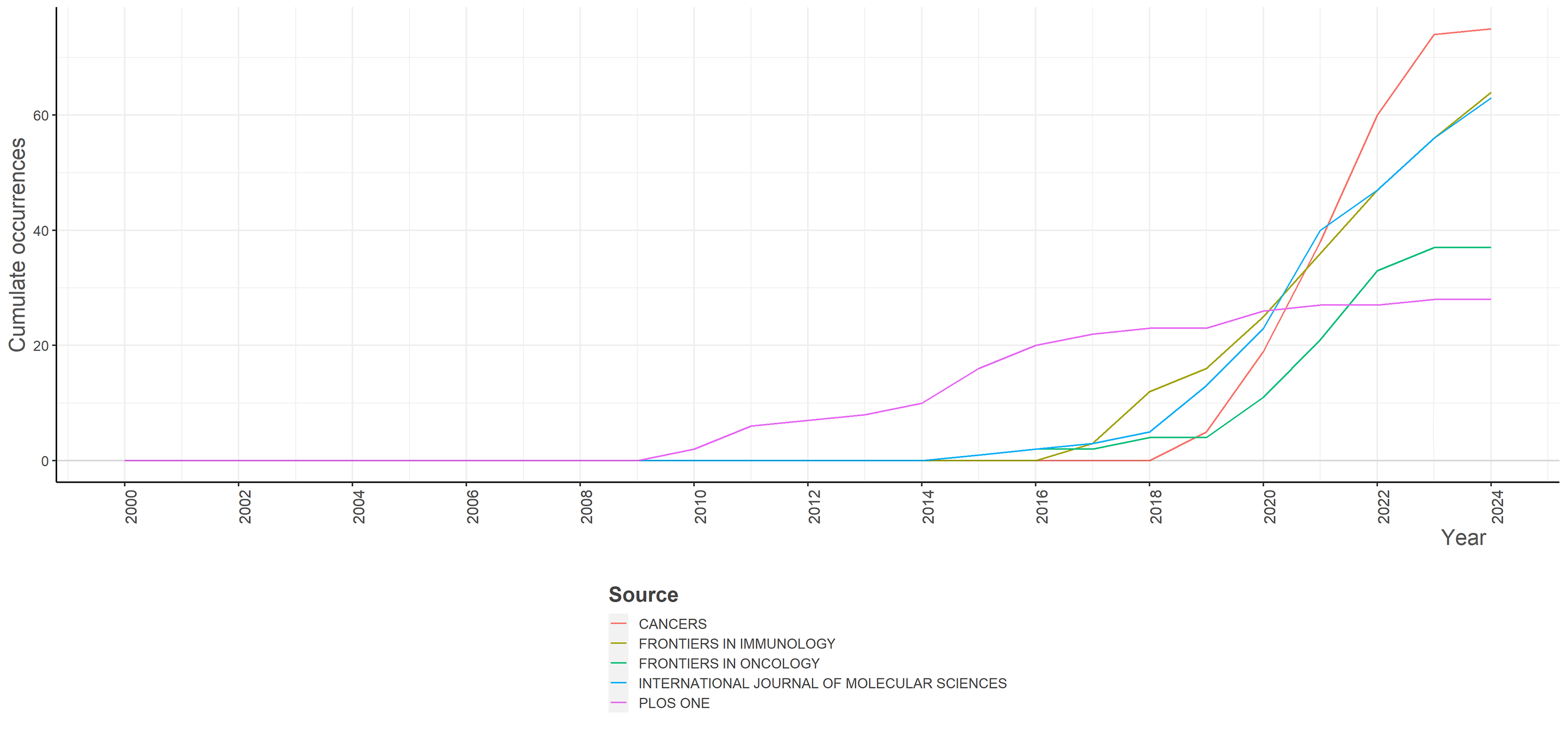


**Figure S4.** The productivity of the five most productive journals has been increasing over time from 2000 to 2024.


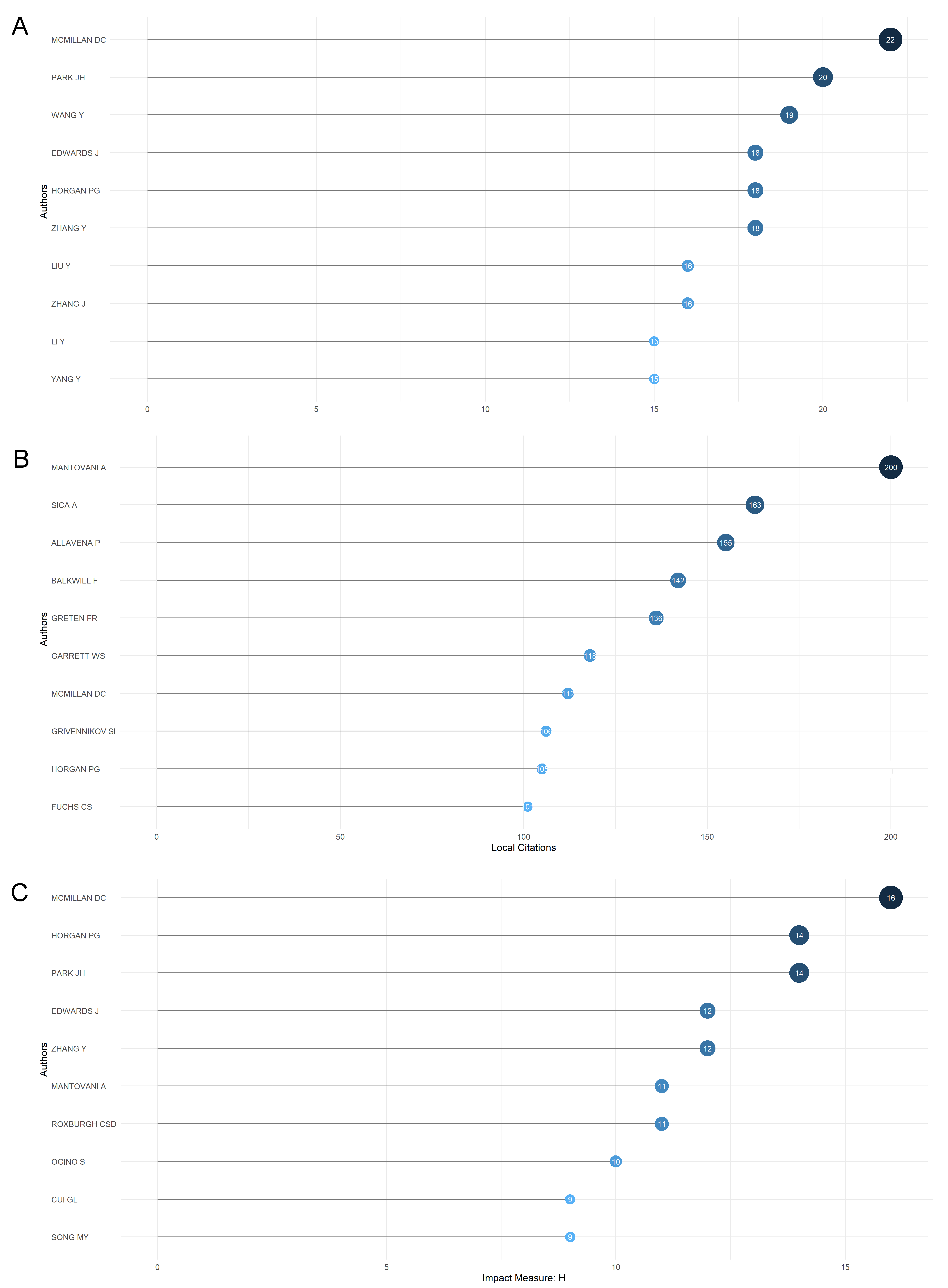


**Figure S5.** The most influential and contributing authors.

1. The top 10 most contributing authors on inflammatory TME with CRC research.
2. The top 10 most cited authors on inflammatory TME with CRC research.
3. the top 10 most locally influential authors on inflammatory TME with CRC research as measured by the H-index.
