# Supplementary material for: Mapping the global intellectual landscape of inflammatory tumor microenvironment in colorectal cancer pathogenesis and prognostic research since this century
Source: Discov Oncol. 2025 Oct 14;16:1884. doi: 10.1007/s12672-025-03524-w (PMC12521722; doi:10.1007/s12672-025-03524-w)
Supplement: Supplementary file 1 — Supplementary Material 1 [file 12672_2025_3524_MOESM1_ESM.zip › supplementary materias/Supplemental file 2.docx]

The original code imported into R package "bibliometrix" is as follows:

install.packages('bibliometrix', dependencies=TRUE)

library(bibliometrix)

biblioshiny()
